# Atrial matrix remodeling in atrial fibrillation patients with aortic stenosis

**DOI:** 10.1186/s12872-020-01754-0

**Published:** 2020-10-31

**Authors:** Mariana Fragão-Marques, I. Miranda, D. Martins, I. Barroso, C. Mendes, A. Pereira-Neves, I. Falcão-Pires, A. Leite-Moreira

**Affiliations:** 1 Cardiovascular Research and Development Center, Faculty of Medicine, University of Porto, Alameda Professor Hernani Monteiro, 4200 Porto, Portugal; 2 Department of Clinical Pathology, São João University Hospital Centre, Porto, Portugal; 3 Department of Biomedicine, Unit of Anatomy, Faculty of Medicine, University of Porto, Porto, Portugal; 4 EPIUnit, Instituto de Saúde Pública, University of Porto, Porto, Portugal

**Keywords:** Atrial fibrillation, Aortic stenosis, Fibrosis, Atrial remodeling, Biomarkers

## Abstract

**Background:**

This study aimed to evaluate atrium extracellular matrix remodeling in atrial fibrillation (AF) patients with severe aortic stenosis, through histological fibrosis quantification and extracellular matrix gene expression analysis, as well as serum quantification of selected protein targets.

**Methods:**

A posthoc analysis of a prospective study was performed in a cohort of aortic stenosis patients. Between 2014 and 2019, 56 patients with severe aortic stenosis submitted to aortic valve replacement surgery in a tertiary hospital were selected.

**Results:**

Fibrosis was significantly increased in the AF group when compared to sinus rhythm (SR) patients (*p* = 0.024). Moreover, cardiomyocyte area was significantly higher in AF patients versus SR patients (*p* = 0.008). Conversely, collagen III gene expression was increased in AF patients (*p* = 0.038). TIMP1 was less expressed in the atria of AF patients. MMP16/TIMP4 ratio was significantly decreased in AF patients (*p* = 0.006). TIMP1 (*p* = 0.004) and TIMP2 (*p* = 0.012) were significantly increased in the serum of AF patients. Aortic valve maximum (*p* = 0.0159) and mean (*p* = 0.031) gradients demonstrated a negative association with serum TIMP1.

**Conclusions:**

Atrial fibrillation patients with severe aortic stenosis present increased atrial fibrosis and collagen type III synthesis, with extracellular matrix remodelling demonstrated by a decrease in the MMP16/TIMP4 ratio, along with an increased serum TIMP1 and TIMP2 proteins.

## Background

Atrial fibrillation (AF) is the most common cardiac arrhythmia with adverse clinical outcomes and diverse pathophysiological background [1]. AF affects approximately 20.9 million men and 12.6 million women worldwide, representing a 1.5-fold and two-fold increase in all-cause mortality, respectively [2].

Aortic stenosis is the most prevalent valvular disease, with aortic valve replacement (AVR) surgery remaining the gold standard treatment for severe symptomatic aortic stenosis, improving both quality of life and overall survival [3]. Owing to ageing demographics, aortic stenosis has been increasing over the past decades, presenting an AF prevalence of 35.6% in a multicentre prospective registry of aortic stenosis patients [4, 5]. However, the pathophysiology of AF in aortic stenosis is poorly understood. AF pathophysiology begins with ectopic firing and re-entry, which depend on several mechanisms: (1) ion channel dysfunction; (2) Ca^2+^-handling abnormalities; (3) structural remodelling; (4) autonomic neural dysregulation. Regarding ion channel dysfunction, cardiomyocytes return to their resting potential after depolarization through an equilibrium between If (pacemaker) and Ik1 (inward rectifier k+) currents, which might be dysfunctional in AF. In addition, early afterdepolarizations (EADs) and delayed afterdepolarizations (DADs), most likely related to Ca^2+^-handling abnormalities, may contribute significantly to AF pathogenesis [6]. Structural and electrical remodelling is core to most forms of AF, particularly in the more permanent ones [7]. Concerning structural remodelling, atrial fibrosis is key and alters cardiomyocyte electrical coupling due to misplacing and changing the structure of connexins, thus inducing fragmented electrical conduction [8]. Evidence suggests atrial remodeling is associated with disease occurrence and progression, which involves specific molecular markers of fibrosis such as Tissue Inhibitor of Metalloproteinase (TIMP) 1, Matrix Metalloproteinase (MMP) 9 and Collagen type 1 Carboxy-terminal Peptide (CICP), with diverse contributions according to different AF subtypes [1]. These biomarkers appear to increase with higher arrhythmia burden [1]. Increased afterload is associated with AF and myocardial fibrosis [5] and while TIMP-1 has been reported to increase, MMP1 was shown to decrease in AF patients’ serum [9]. Conversely, atrium TIMP-4 in AF patients with rheumatic heart disease was lower when compared with Sinus Rhythm (SR) patients, while MMP-2, type I, and type II collagen were increased in the AF group [10].

This is the first study aimed to characterize atrial fibrosis in AF patients with aortic stenosis submitted to AVR surgery, as well as evaluate the gene expression profile of several extracellular matrix proteins and quantify differentially expressed targets in the serum of AF patients.

## Methods

This study aimed to evaluate atrium extracellular matrix remodeling in AF patients with severe aortic stenosis, through histological fibrosis quantification and extracellular matrix gene expression analysis of an array of genes: collagen I and III, TIMP 1, TIMP2, TIMP4, MMP 2, MMP9, MMP16, and TGF-β1. Serum quantification of target extracellular matrix proteins was performed to establish potential AF biomarkers (TIMP1 and TIMP2).

### Study design

This study represents a posthoc analysis of a prospective study on a cohort of aortic stenosis patients. Between 2014 and 2019, 56 patients with severe aortic stenosis submitted to AVR surgery in a tertiary hospital were selected. Their baseline clinical and echocardiography data were recorded, and serum and atrium samples were collected. Reoperations and emergent cases were excluded, as well as patients with severe aortic regurgitation. The recruited patients were then grouped according to their cardiac rhythm status: AF or SR (N = 15 and N = 42, respectively) (*see below*).

The investigation conforms with the principles outlined in the Declaration of Helsinki. The protocol was approved by the institution’s ethics committee and data confidentiality was assured. All participants gave written informed consent.

### Data collection

Chronic Kidney Disease was considered when baseline creatinine levels exceeded 1.5 mg/dL. New York Heart Association (NYHA) functional classification was used to characterize heart failure symptoms. Coronary Artery Disease (CAD) was considered when previous myocardial infarction was reported and/or invasive coronary angiography reported stenosis > 50%. Cerebrovascular disease was defined based on a history of stroke or transitory ischemic attack or a reported stenosis > 50% on carotid doppler ultrasonography. Smoking was considered when patients reported being active smokers or past smokers within less than a year of cessation. Patients underwent an echocardiographic evaluation performed by experienced operators up to 6 months before surgery. Cardiac chamber dimensions and volumes were measured as recommended and systolic function was accessed by evaluation of left ventricular ejection fraction, using the modified Simpson rule from biplane 4 and 2 chamber views [11]. Reduced ejection fraction was considered below 40%, according to the European Society of Cardiology guidelines [12] Left atrium enlargement was considered when left atrium diameter was equal or superior to 40 mm.

As previously mentioned, patients were divided into SR and AF groups. All patients had an electrocardiogram (EKG) performed up to 6 months prior to surgery and AF was defined according to international guidelines as absolutely irregular RR intervals and no discernible, distinct P waves, with an episode lasting at least 30 s being the threshold for diagnostic purposes [2]. Definitions of paroxysmal, persistent, long-standing persistent or permanent AF followed the European Society of Cardiology guidelines. Two patients presented with paroxysmal AF (13.3%), 8 with permanent AF (53.3%) and 5 with persistent AF (33.3%) [2].

### Biopsy and serum sample collection

During surgery, an atrium appendix myocardial biopsy was collected, and the sample was either immediately fixed in formalin (formalin solution, neutral buffered, 10%) and processed for histological analysis or flash-frozen in liquid nitrogen and stored at -80ºC for posterior gene expression analysis. Whole blood samples were collected at the same timepoint and immediately centrifuged at 3000 rpm for 10 min; serum was stored at – 80 °C for further Enzyme-Linked ImmunoSorbent Assay (ELISA).

### Histomorphometric quantification

All samples were processed using *Leica Histocore Pearl* automatic processor. A total of 29 (SR N = 19, AF N = 10) and 18 patients (SR N = 13, AF = 5) were used to assess fibrosis and cardiomyocyte cross-sectional area, respectively. Paraffin inclusion was performed in *Leica Histocore Arcardia Embedding System*. Sections of 3 µm were cut and stained with hematoxylin–eosin (Harris hematoxylin and alcoholic eosin) for assessing cardiomyocyte cross-sectional area using *Leitz Wetzlar-Dialux 20* microscopy and coupled camera (*Olympus XC30*). At least 60 cardiomyocytes were counted using the 250 × amplification; fibrosis was assessed using 3 µm atrial sections stained with Red Sirius and quantification was performed with Image-Pro Plus in 8 fields per sample using the 100 × amplification (results presented as percentage of the total area of each section).

### Gene expression analysis

A total of 19 patients (SR N = 11, AF N = 8) were evaluated for the gene expression of several extracellular matrix proteins: collagen I and III, TIMP1, TIMP2, TIMP4, MMP2, MMP9, MMP16 and Transforming Growth Factor β1 (TGF-β1). RNA extraction was performed using a TRIzol protocol following the manufacturer's instructions. Briefly, tissue cells were disrupted in TRIizol reagent using 1.4 mm beads (Minilys, Bertin). After adding chloroform and centrifugation at 14,000 rpm, at 4 °C, for 15 min, RNA was extracted and precipitated in isopropanol. RNA was washed in 70% ethanol and resuspended in Tris 10 mM. RNA concentration and integrity were assessed both in the Nanodrop and by electrophoresis. cDNA (100 ng/µL) was synthesized using the SensiFAST™ cDNA Synthesis Kit (Bioline). Reactions were performed in a Bio-Rad T100 thermal cycler using the following protocol: 10 min of primer annealing at 25 °C followed by reverse transcription for 15 min at 42 °C, followed by 5 min at 85 °C to inactivate reverse transcriptase. Quantitative polymerase chain reaction (qPCR) reactions were run in triplicate in 10 µL containing the following: 1 µL sample cDNA, 5 µL 2 × SensiFAST™ SYBR Hi-ROX Mix (Bioline), and 0.4 µL primers. RT-qPCR reactions were performed using the PikoReal™ Real-Time PCR System (Thermo Scientific™) using the following protocol: polymerase was activated for 3 min at 95 °C followed by 40 cycles of denaturation for 15 s at 95 °C, annealing for 30 s at 60 °C, and extension for 30 s at 72 °C. Thereafter, melting curve analysis from 65 to 95 °C in 0,5 °C increments was performed. Prior to gene expression quantification using the 2^−ΔCT^ method, PCR efficiency of each gene including the internal control gene (18s RNA) was determined and it was assured that they were identical. Primer sequences are presented in Table 1.

**Table 1:** Primers used in this study

Primer name	Primer sequences (5′ to 3′)
HsCOL1A1_F1	CTCTGGTCCTCGTGGTCT
HsCOL1A1_R1	CCCCATCATCTCCATTCTTTCC
HsCOL3A1_F1	GGTGCTAATGGTGCTCCT
HsCOL3A1_R1	TCCTTGCCATCTTCGCCTTT
HsMMP2_F	TTGGTGGGAACTCAGAAGGT
HsMMP2_R	GTCATCATCGTAGTTGGCTGTG
HsMMP9_F	CTGCCACTTCCCCTTCATC
HsMMP9_R	GGTCGTCGGTGTCGTAGT
HsMMP16_F	TGACCCCAGAATGTCAGTGC
HsMMP16_R	GGGGCTTCTTCATCCAGTCAA
HsTIMP1_F	CTGTTGGCTGTGAGGAATGC
HsTIMP1_R	AGGTGACGGGACTGGAAG
HsTIMP2_F	GAGCACCACCCAGAAGAAG
HsTIMP2_R	CAGTCCATCCAGAGGCAC
HsTIMP4_F	GACCCTGCTGACACTGAAAA
HsTIMP4_R	CTTCTGGCTGTTGGCTTCTA
HsTGFB1_F	GAAGAACTGCTGCGTGCG
HsTGFB1_R	GTGTCCAGGCTCCAAATGT
Hs18s_F2	GACTCAACACGGGAAACCTC
Hs18s_R2	CCAGACAAATCGCTCCACC

Primer name and respective base-pair sequence

Target gene expression was normalized to the 18S gene. Initial mRNA expression data were logarithm transformed using the 2^−ΔC^_T_ method [13].

### Serum quantification

After gene expression analysis, a total of 24 patients (SR N = 17, AF N = 7) were selected for assessing serum quantification of proteins whose genes were shown to be increased by reverse transcription (RT)-PCR in AF patients (TIMP1 and 2, see below). ELISA assays were performed following the manufacturer’s instructions (Abcam, ab187394 Human TIMP-1 SimpleStep ELISA Kit, and ab100653 TIMP2 Human ELISA kit).

### Statistical analysis

IBM SPSS Statistics version 25 was used for all statistical analyses. Categorical variables were presented as percentages and continuous variables as mean and standard deviation (sd). Categorical variables were analysed using the Chi-squared test or Fisher’s exact test when appropriate and continuous variables were analysed with a t-test for independent samples. Linear regression was performed to correlate continuous variables and results are presented as β coefficients, 95% confidence intervals (CI), and *p* values. Statistical significance was considered when *p* < 0.05.

## Results

### Patient demographics

Participants had a mean age of 70.85 ± 10.00 years, with males representing 49.2% of patients. The most common comorbidity was hypertension, representing 64.4% of patients, while 20.3% had type 2 diabetes mellitus. Smoking habits were present in 8.5% of participants, although there was no chronic kidney disease in this cohort. Only 2 patients had a previous acute myocardial infarction (3.4%), though 15.3% revealed coronary artery disease on coronary angiography. Moreover, 2 patients had a history of cerebrovascular disease (3.4%). Symptoms of heart failure (NYHA > I) were present in 55.9% of patients, with NYHA class II being the most prevalent stage (54.2%); 15.3% of patients suffered from angina, while only 5.1% had complaints of syncope or lipothymia. Solely one patient had decreased left ventricular ejection fraction. Nonetheless, 64.4% had increased left atrium diameter. Aortic valve stenosis was severe, with a mean aortic valve maximum gradient of 83.04 ± 20.47 mmHg, an aortic valve mean gradient of 51.32 ± 12.36 mmHg and an aortic valve area of 0.76 ± 0.16 cm^2^.

Concerning SR and AF patient subgroups, AF patients were older (AF 75.47 ± 7.71 vs SR 68.98 ± 10.23 years, *p* = 0.023). There were no other differences between groups regarding demographics, comorbidities, and echocardiographic data (Table 2).

**Table 2:** Patient demographics

Variable [n (%)]	SR (N = 42)	AF (N = 15)	*p* value
Age, years [mean ± SD]	68.98 ± 10.23	75.47 ± 7.71	**0.023**
Gender (male)	21 (50.0)	8 (47.1)	0.838
Smoking	5 (11.9)	0 (0)	0.308
Diabetes mellitus (type 2)	7 (16.7)	5 (29.4)	0.299
Hypertension	28 (66.7)	10 (62.5)	0.765
Previous AMI	2 (4.8)	0 (0)	1.000
Coronary artery disease	7 (17.1)	2 (12.5)	1.000
Cerebrovascular disease	1 (2.4)	1 (6.3)	0.479
NYHA class > I	23 (63.9)	10 (66.7)	0.850
Angina	5 (13.9)	4 (28.6)	0.245
Syncope/lipothymia	2 (5.6)	1 (7.1)	1.000
Ejection fraction	0 (0)	1 (7.1)	0.280
Left atrium dilation	26 (81.3)	12 (92.3)	0.654
AoV MaxG, mmHg [mean ± SD]	84.90 ± 20.23	78.93 ± 21.14	0.371
AoV MeanG, mmHg [mean ± SD]	52.36 ± 11.95	48.86 ± 13.41	0.380
Aov area, cm^2^ [mean ± SD]	0.76 ± 0.16	0.75 ± 0.15	0.784

*AMI* acute myocardial infarction, *NYHA* New York Heart Association Functional Capacity Classification, *AoV* aortic valve, MaxG maximum gradient

### Atrial fibrillation

#### Histomorphometric analysis

**Fig. 1:**
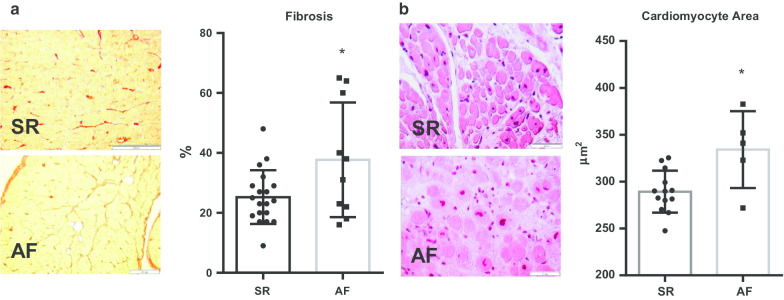
**a** Fibrosis quantification by Sirius red stain—data and representative images of both AF and SR patient subgroups. **b** Cardiomyocyte area quantification by hematoxylin–eosin stain—data and representative images of AF and SR patients. *p > 0.05

Fibrosis was significantly increased in the AF group when compared to SR patients, with a mean ± SD percent fibrosis of 38 ± 6% and 25 ± 2%, respectively (*p* = 0.024). Moreover, cardiomyocyte area was significantly higher in AF patients versus SR patients—334.1 ± 18.31 µm^2^ versus 289.3 ± 6.18 µm^2^ (*p* = 0.008) (Fig. 1).

#### Extracellular matrix gene expression analysis

**Fig. 2:**
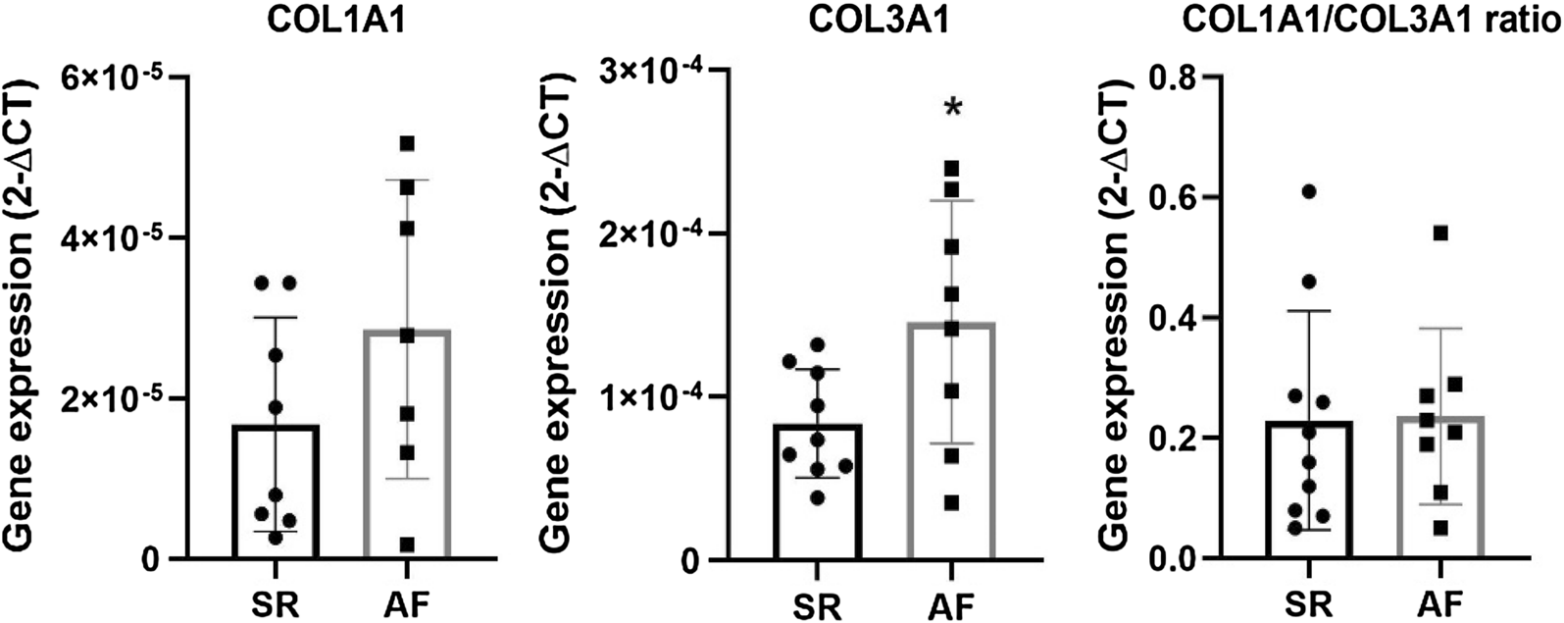
Atrial gene expression of collagen type I, type III and respective ratio in both SR and AF patients. *p < 0.05

Collagen gene expression was evaluated to explore the differences in fibrosis observed through histologic examination. Atrial expression of collagen I was similar between groups (2^−ΔCT^): AF 2.86 × 10^–5^ ± 7.03 × 10^–6^ versus SR 1.67 × 10^–5^ ± 4.71 × 10^–6^ (*p* = 0.176). Conversely, collagen III gene expression was increased in AF patients: 1.46 × 10^–4^ ± 2.62 × 10^–5^ versus 8.39 × 10^–5^ ± 1.11 × 10^–5^ (*p* = 0.038). The collagen ratio I/III was, nonetheless, similar between AF and SR patients: 0.24 ± 0.05 versus 0.23 ± 0.06 (*p* = 0.928). Data is depicted in Fig. 2.

**Fig. 3:**
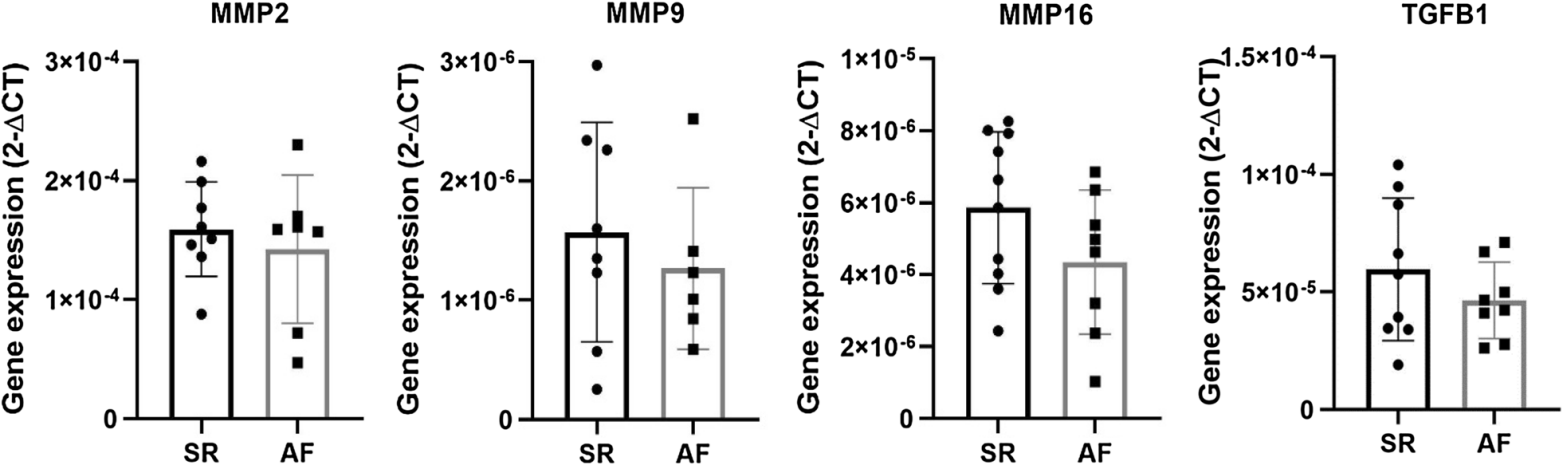
Atrial gene expression of MMP2, MMP9, MMP16 and TGFβ in both SR and AF patients

**Fig. 4:**
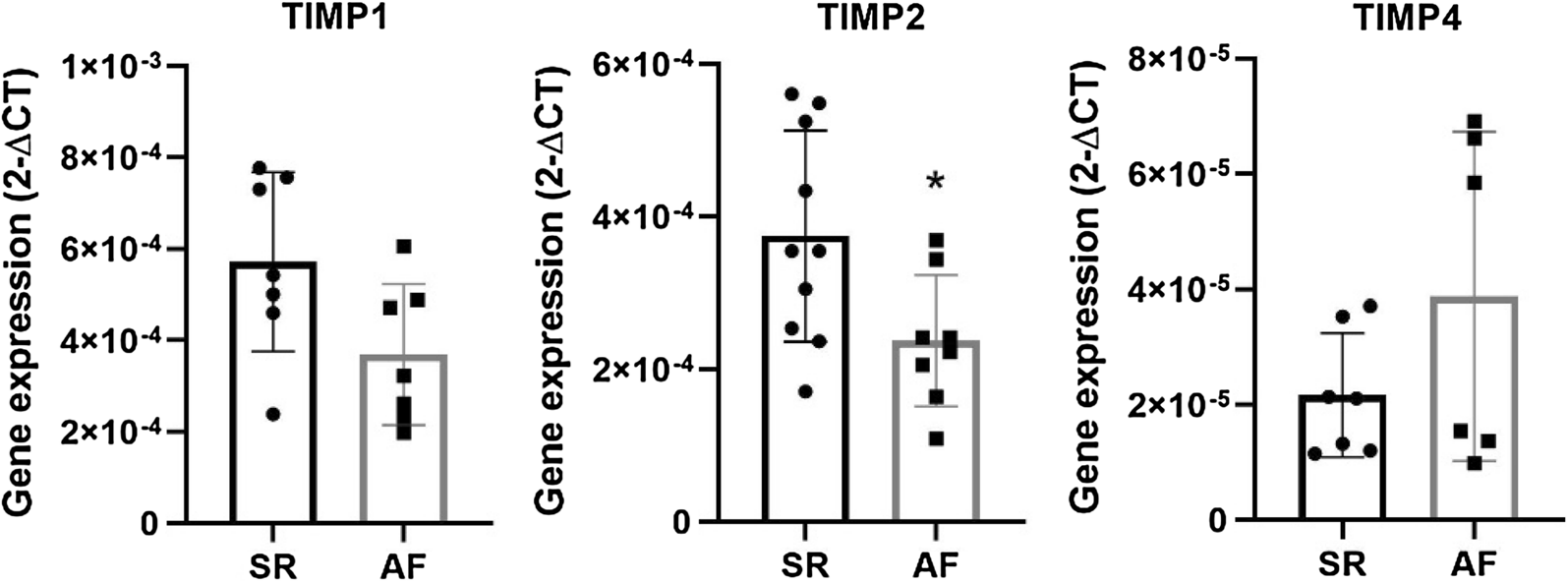
Atrial gene expression of TIMP1, TIMP2 and TIMP4 in both SR and AF patients. *p < 0.05

A further analysis on extracellular matrix genes was performed, with several MMPs and TIMPs tested for their atrial expression. MMP2 expression analysis showed no differences between groups: AF 1.42 × 10^–4^ ± 2.35 × 10^–5^ versus SR 1.59 × 10^–4^ ± 1.40 × 10 ^−5^ (*p* = 0.536). MMP9 and MMP16 were similar between patients, showing no differential atrial expression: 1.27 × 10^–6^ ± 2.77 × 10^–7^ versus 1.57 × 10^–6^ ± 3.25 × 10^–7^ (*p* = 0.509) and 4.35 × 10^–6^ ± 7.08 × 10^–7^ versus 5.86 × 10^–6^ ± 6.66 × 10^–7^ (*p* = 0.141), respectively. In addition, TGFβ1 gene expression analysis was similar between patient groups: 4.65 × 10^–5^ ± 5.75 × 10^–6^ versus 5.96 × 10^–5^ ± 1.01 × 10^–5^ (*p* = 0.291) (Fig. 3). On the other hand, TIMP1 was less expressed in the atria of AF patients: 3.69 × 10^–4^ ± 5.82 × 10^–5^ versus 5.72 × 10^–4^ ± 7.41 × 10^–5^ (*p* = 0.052). TIMP2 presented similar results, demonstrating a significantly decreased expression in the AF group: 2.37 × 10^–4^ ± 3.04 × 10^–5^ versus 3.75 × 10^–4^ ± 4.37 × 10^–5^ (*p* = 0.026). Conversely, TIMP4 did not show differences between patients: 3.88 × 10^–5^ ± 1.17 × 10^–5^ versus 2.17 × 10^–5^ ± 4.06 × 10^–6^ (*p* = 0.167) (Fig. 4).

**Fig. 5:**
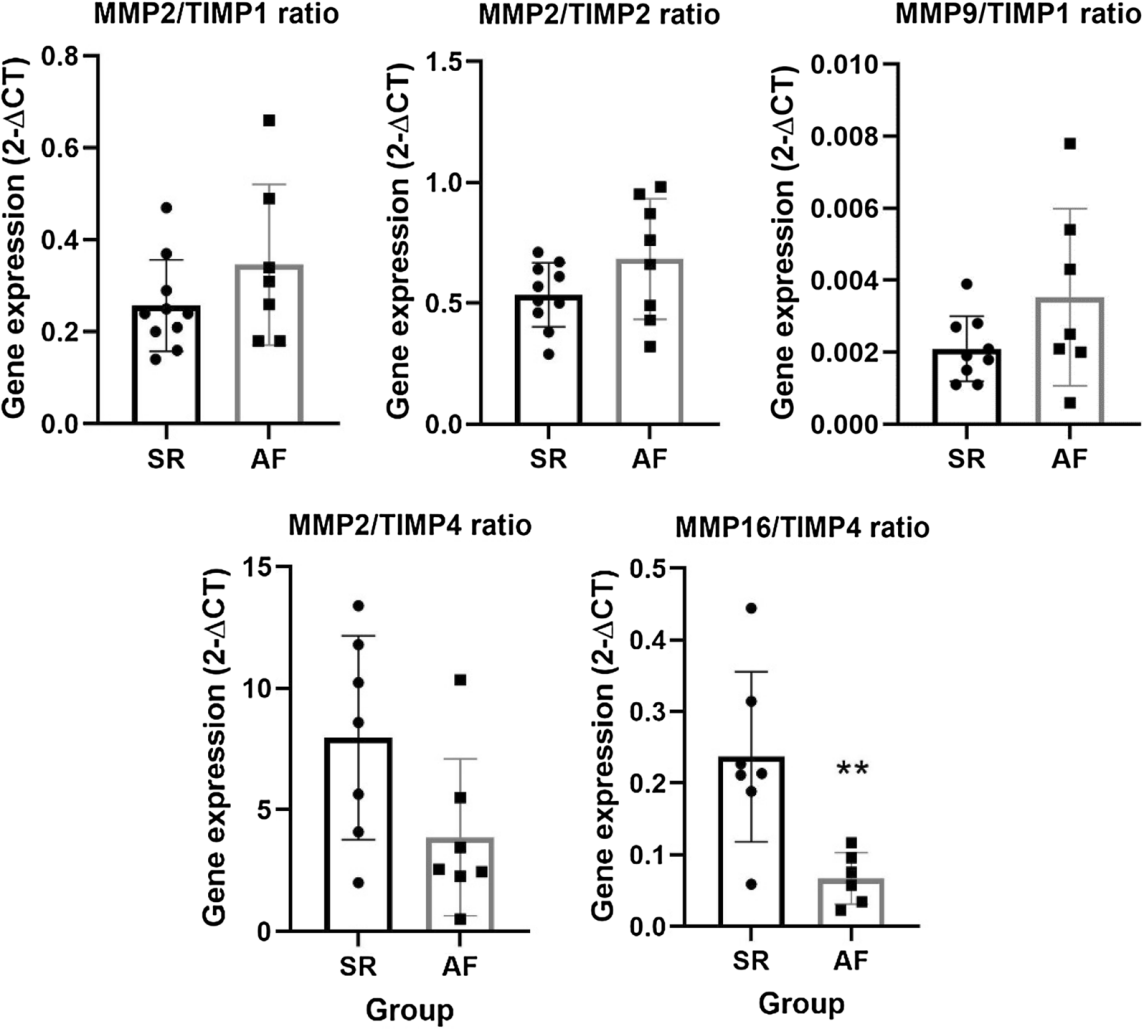
Atrial gene expression of MMP2/TIMP1, MMP2/TIMP2, MMP9/TIMP1, MMP2/TIMP4 and MM16/TIMP4 ratios in both SR and AF patients. **p < 0.001

Moreover, well recognized ratios such as MMP2/TIMP1, MMP2/TIMP2 and MMP9/TIMP1 were tested for their relationship with AF. MMP2/TIMP1 did not present differences between groups, with a ratio of 0.35 ± 0.07 in AF patients and 0.26 ± 0.03 in SR patients (*p* = 0.201). Likewise, both the MMP2/TIMP2 and MMP9/TIMP1 ratios were similar between groups—0.68 ± 0.09 versus 0.53 ± 0.04 (*p* = 0.123) and 3.53 × 10^–3^ ± 9.30 × 10^–4^ versus 2.10 × 10^–3^ ± 3.02 × 10^–4^ (*p* = 0.128). (Fig. 5). MMP2/TIMP4 and MMP16/TIMP4 ratios were also tested, with the former demonstrating a tendency towards significance (3.87 ± 1.22 vs 7.96 ± 1.58, *p* = 0.063), while the latter was significantly decreased in AF patients (0.07 ± 0.02 vs 0.24 ± 0.05, *p* = 0.006).

#### Serum TIMP1 and TIMP2

**Fig. 6:**
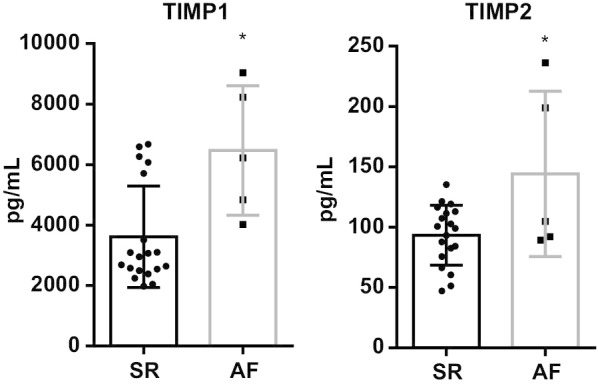
Serum TIMP1 and TIMP2 proteins in both SR and AF patients. *p < 0.05

The counterpart proteins to the altered gene expression in AF patients (TIMP1 and TIMP2, *p* = 0.052 and *p* = 0.026, respectively) were quantified in the serum of both AF and SR patient groups. TIMP1 was significantly increased in the serum of AF patients: AF 6472.0 ± 958.9 pg/mL versus SR 3612.0 ± 385.4 pg/mL (*p* = 0.004). Similarly, TIMP2 was increased in the AF patient group: 144.2 ± 30.6 pg/mL versus 93.4 ± 5.7 pg/mL (*p* = 0.012) (Fig. 6).

### Aortic stenosis severity

The severity of aortic stenosis was evaluated through aortic valve maximum and mean gradients, as well as aortic valve area. These predictors were correlated with histological parameters and extracellular matrix gene expression and serum proteins. Results are presented in Table 3.

**Table 3:** Linear regressions comparing aortic stenosis severity with extracellular matrix remodeling

Variable	B coefficient	95% CI	*p* value
**AoMaxG**			
*Cardiomyocyte Area*	0.089	[− 0.069 to 0.248]	0.250
*Fibrosis*	− 8.431	[− 184.602 to 167.741]	0.920
*COL1A1*	− **493,530.998**	**[**− **929322.424** to − **57739]**	**0.029**
*COL3A1*	− 106,482.445	[− 240598.920 to 27,634.031]	0.110
*COL1A1/3A1 Ratio*	− **88.731**	**[**− **165.756** to − **11.706]**	**0.027**
*MMP2*	− 27,782.426	[− 170179.428 to 114,614.396]	0.680
*MMP9*	− 595,526.093	[− 1429715.173 to 238,662.988]	0.147
*MMP16*	1,691,707.088	[− 3869174.848 to 7,252,589.024]	0.523
*TIMP1*	14,457.016	[− 7526.012 to 36,440.044]	0.179
*TIMP2*	5262.861	[− 85597.990 to 96,123.712]	0.902
*TIMP4*	− 47,199.665	[− 445150.640 to 350,751.311	0.799
*MMP2/TIMP1 Ratio*	− 79.725	[− 159.870 to 0.419]	0.051
*MMP2/TIMP2 Ratio*	− 30.903	[− 94.861 to 33.056]	0.316
*MMP9/TIMP1 Ratio*	− 330.433	[− 732.257 to 71.391]	0.099
*MMP2/TIMP4 Ratio*	− 0.345	[− 2.205 to 1.516]	0.691
*MMP16/TIMP4 Ratio*	7.394	[− 69.324 to 84.111]	0.836
*Serum TIMP1*	− **0.004**	**[**− **0.006** to − **0.001]**	**0.015**
*Serum TIMP2*	0.028	[− 0.235 to 0.292]	0.822
**AoMG**			
*Cardiomyocyte Area*	0.038	[− 0.059 to 0.135]	0.421
*Fibrosis*	9.331	[− 93.022 to 111.684]	0.850
*COL1A1*	− **248,924.745**	**[**− **492536.116** to − **5313.373]**	**0.046**
*COL3A1*	− 55,243.442	[− 128681.989 to 18,195.105]	0.128
*COL1A1/3A1 Ratio*	− **44.441**	**[**− **87.659** to − **1.224]**	**0.045**
*MMP2*	− 8256.311	[− 85857.707 to 69,345.085]	0.822
*MMP9*	− 273,834.048	[− 737776.442 to 190,108.346]	0.225
*MMP16*	1,163,548.388	[− 1821928.808 to 4,149,025.584]	0.415
*TIMP1*	9237.603	[− 2320.881 to 20,796.086]	0.108
*TIMP2*	8692.535	[− 40342.265 to 57,727.334]	0.708
*TIMP4*	− 20,738.463	[− 241638.087 to 200,161.161]	0.840
*MMP2/TIMP1 Ratio*	− **44.416**	**[**− **87.457** to − **1.375]**	**0.044**
*MMP2/TIMP2 Ratio*	− 20.424	[− 54.401 to 13.554]	0.217
*MMP9/TIMP1 Ratio*	− 157.431	[− 381.331 to 66.470]	0.153
*MMP2/TIMP4 Ratio*	− 0.215	[− 1.244 to 0.815]	0.655
*MMP16/TIMP4 Ratio*	0.796	[− 41.824 to 43.416]	0.968
*Serum TIMP1*	− **0.002**	**[**− **0.004** to − **0.0002]**	**0.031**
*Serum TIMP2*	0.002	[− 0.166 to 0.169]	0.983
**AVA**			
*Cardiomyocyte Area*	− **0.001**	**[**− **0.002** to − **2.6E**−**5]**	**0.043**
*Fibrosis*	− 0.628	[− 1.749 to 0.493]	0.251
*COL1A1*	**4045.908**	**[564.094** to **7527.721]**	**0.026**
*COL3A1*	999.124	[− 42.261 to 2040.509]	0.059
*COL1A1/3A1 Ratio*	**0.684**	**[0.050** to **1.319]**	**0.037**
*MMP2*	31.793	[− 1153.160 to 1216.745]	0.954
*MMP9*	2748.383	[− 4536.323 to 10,033.089]	0.427
*MMP16*	− 16,518.724	[− 64280.280 to 31,242.833]	0.466
*TIMP1*	− 65.689	[− 255.398 to 124.020]	0.465
*TIMP2*	− 118.969	[− 861.135 to 623.197]	0.733
*TIMP4*	− 1791.725	[− 4921.305 to 1337.855]	0.231
*MMP2/TIMP1 Ratio*	0.089	[− 0.678 to 0.855]	0.806
*MMP2/TIMP2 Ratio*	0.309	[− 0.225 to 0.843]	0.231
*MMP9/TIMP1 Ratio*	1.551	[− 2.011 to 5.114]	0.361
*MMP2/TIMP4 Ratio*	0.009	[− 0.006 to 0.023]	0.200
*MMP16/TIMP4 Ratio*	0.281	[− 0.326 to 0.889]	0.326
*Serum TIMP1*	8.697E−6	[2.4E−5 to 4.1E−5]	0.577
*Serum TIMP2*	− 0.001	[− 0.004 to 0.001]	0.303

Results in bold represent *p* < 0.05

*AoMaxG* aortic valve maximum gradient, *AoMG* aortic valve mean gradient, *AVA* aortic valve area, *CI* confidence interval, *MMP* matrix metalloproteinase, *TIMP* tissue inhibitor of metalloproteinase

#### Histomorphometric analysis

**Fig. 7:**
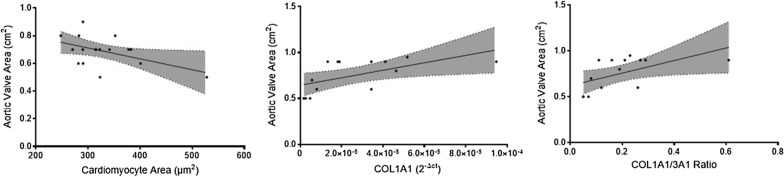
Linear regression between Aortic Valve Area and atrial cardiomyocyte area and collagen type I and III gene expression. (All *p* < 0.05)

Neither the aortic valve maximum gradient (*p* = 0.920 and *p* = 0.250) nor its mean gradient (*p* = 0.850 and *p* = 0.421) correlated with fibrosis or atrial cardiomyocyte size, respectively. However, aortic valve area was negatively correlated with cardiomyocyte area—B coefficient [95% CI] − 0.001 [− 0.002 to − 2.6 × 10^–5^], *p* = 0.043 (Fig. 7). In addition, fibrosis was not correlated with aortic valve area (*p* = 0.251).

#### Extracellular matrix gene expression analysis

**Fig. 8:**
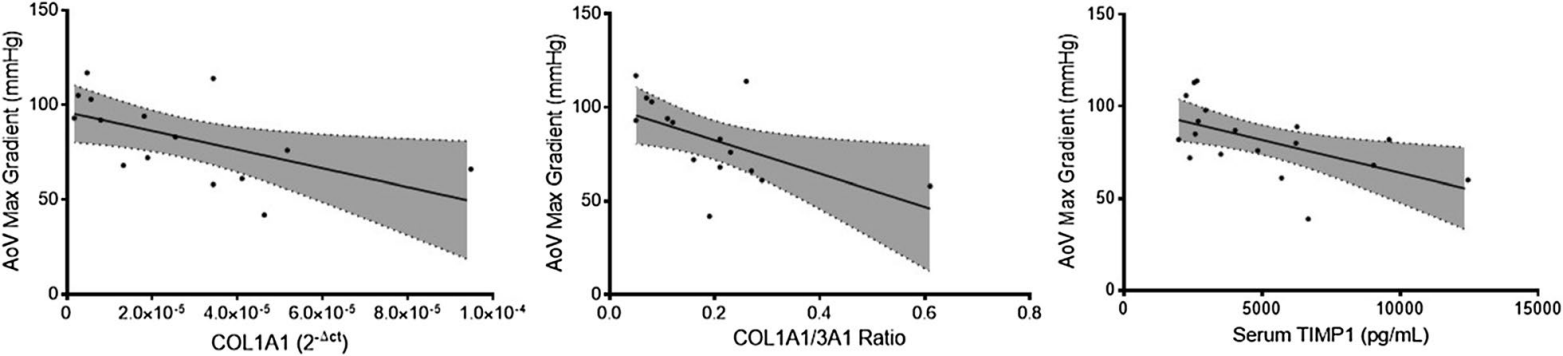
Linear regression between aortic valve maximum gradient and collagen type I gene expression, collagen type I/III ratio gene expression and serum TIMP1 protein levels. (All *p* < 0.05)

Aortic valve maximum gradient was inversely correlated with the expression of COL1A1 gene (B − 493,531.00 [− 929322.42 to − 57,739.00], *p* = 0.029), as well as the COL1A1/3A1 gene expression ratio (B − 88.73 [− 165.76 to − 11.71], *p* = 0.027). (Fig. 8) Furthermore, MMP2/TIMP1 ratio presented a tendency towards significance, with a B coefficient of − 79.73 [− 159.87 to 0.42], *p* = 0.051. The remaining extracellular genes were not correlated with aortic valve maximum gradient (Table 3).

**Fig. 9:**
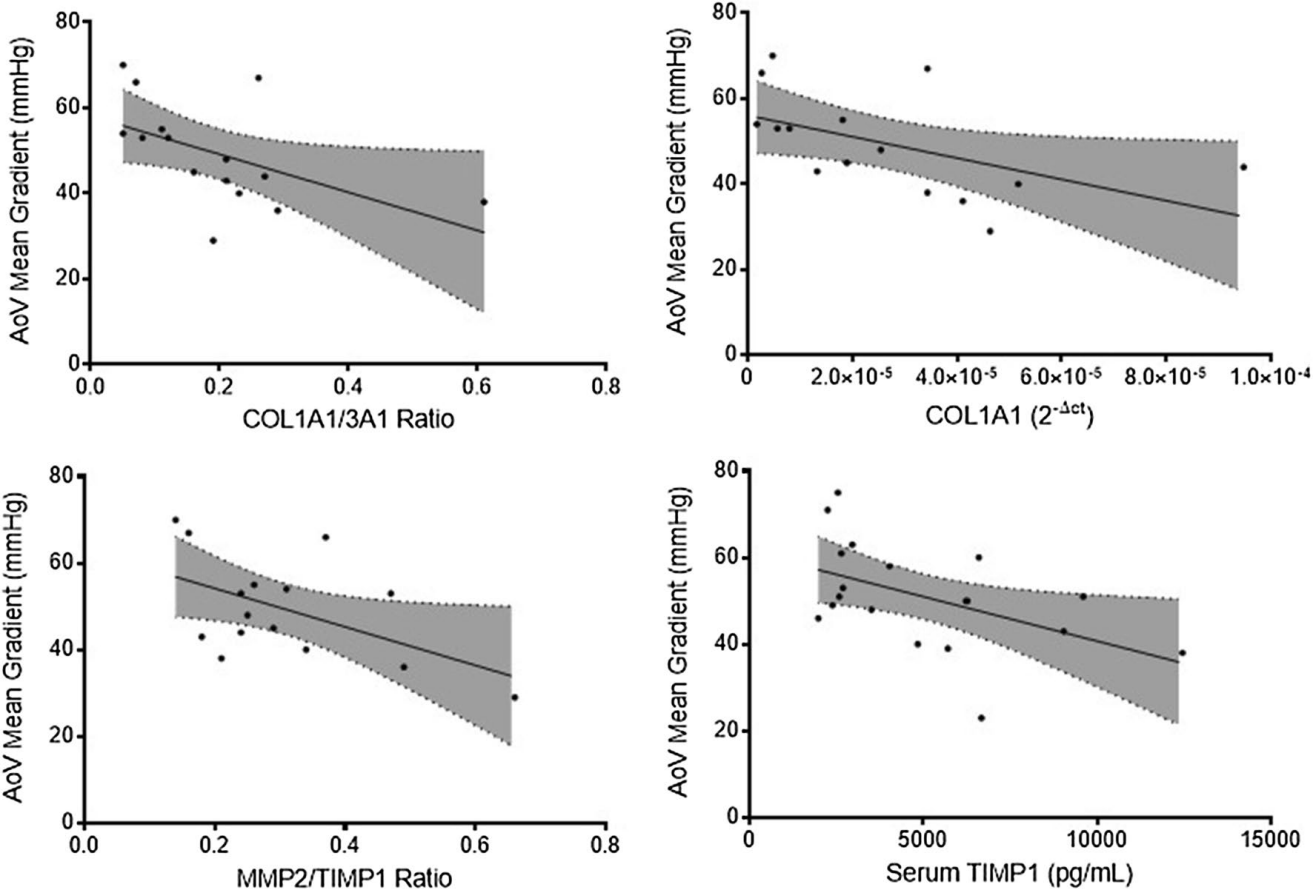
Linear regression involving aortic valve mean gradient and collagen type I gene, collagen type I/III ratio gene expression, MMP2/TIMP1 gene expression ratio and serum TIMP1 protein quantification. (All *p* < 0.05)

Similarly, aortic valve mean gradient was negatively correlated with COL1A1 expression (B − 248,924.75 [− 492536.12 to − 5313.37], *p* = 0.046), COL1A1/3A1 ratio (B − 44.44 [− 87.66 to − 1.22], *p* = 0.045) and MMP2/TIMP1 ratio (B − 44.42 [− 87.46 to − 1.38], *p* = 0.044). (Fig. 9) Other matrix genes remained similar regardless of aortic valve mean gradient (Table 3).

Additionally, aortic valve area presented a positive correlation with the gene expression of COL1A1 (B 4045.91 [564.09–7527.72], *p* = 0.026) and COL1A1/3A1 ratio (B 0.68 [0.05–1.32], *p* = 0.037) (Fig. 7), although other extracellular matrix genes presented no relation with valve area (Table 3).

#### Serum TIMP1 and TIMP2

Aortic valve maximum gradient demonstrated a negative association with serum TIMP1, with a B coefficient of − 0.004 [− 0.006 to − 0.001], *p* = 0.015. (Fig. 8) Likewise, aortic valve mean gradient was negatively correlated with serum TIMP1—B − 0.002 [− 0.004 to − 0.0002], *p* = 0.031. (Fig. 9) No other associations were observed regarding aortic valve function and serum TIMP1 and TIMP2 (Table 3).

## Discussion

This is the first study evaluating atrial remodelling in aortic stenosis patients with chronic AF, both fibrosis quantification and target extracellular matrix protein gene expression analysis. AF patients were older, had increased cardiomyocyte area and atrial fibrosis on histologic quantification, increased collagen type III gene expression, as well as decreased TIMP1 and TIMP2 gene expression. Moreover, MMP16/TIMP4 ratio was decreased in AF patients. Serum TIMP1 and TIMP2 proteins were both increased in the AF patient subgroup.

Aortic stenosis patients with AF were older than their SR counterparts. Age is the most common and consistently described risk factor for AF, regardless of the cardiovascular disease background [4, 14]. Patients with AF had increased fibrosis, as demonstrated by histology sections, as well as increased cardiomyocyte size, suggested by the higher cell area. There is extensive evidence on the role of atrial fibrosis in sustaining the arrhythmia [15], although studies on aortic stenosis are scarce. Studies on animal models with increased afterload but absent hypertension suggest atrial remodeling increases AF inducibility through atrial myocardium fibrosis [16]. On the other hand, fibrosis evaluated by cardiac magnetic resonance imaging suggests a correlation with atrial fibrillation in hypertrophic cardiomyopathy [17]. Aortic stenosis causes pressure overload in both the ventricle and atrium, thus stimulating fibroblast proliferation which leads to cardiac fibrosis [18]. Fibroblast-cardiomyocyte coupling might slow conduction, therefore promoting re-entrance mechanisms, as well as enhance phase 4 depolarization, inducing ectopic impulse generation [19]. This cell-to-cell interaction depends on the degree of coupling, the number of coupled fibroblasts to each cardiomyocyte, and the relative size of cardiomyocytes versus fibroblasts [20]. Furthermore, the increased cardiomyocyte cell area in AF patients might correspond to a glycogen accumulation, with depletion of contractile material, as demonstrated in a study of sustained AF in goats with a substantial proportion of atrial myocytes with marked ultrastructure changes, including loss of myofibrils and accumulation of glycogen [21]. Studies on dogs with mitral valve stenosis have reported atrial cardiomyocyte hypertrophy with decreased myofibrils [21, 22]. Despite the absence of studies on aortic stenosis patients, it has been reported similar degenerative cardiomyocyte changes in patients submitted to cardiac surgery, which correlate with atrial size and pressure, along with diastolic dysfunction [23].

In addition to the increase in atrial fibrosis in histology sections, mRNA expression of collagen type III was higher in AF patients, although collagen I and collagen I/III ratio did not present differences between groups. Zhang et al. reported an increase in atrial mRNA expression of collagen I and III in AF patients with rheumatic heart disease [24]. Likewise, Cao et al. reported a similar increase in patients with permanent AF undergoing valvular replacement [25]. Conversely, Yoshihara et al. found a decrease in mRNA expression of both collagen type I and III in patients with AF submitted to Kosakai's modified maze procedure [26]. The present study focused on AF patients with aortic stenosis submitted to valve replacement, which could explain the isolated increase in type III collagen gene expression between patient groups.

Regarding atrial extracellular matrix gene expression, TGF-β1 was similar between groups. TGFβ1 is an established positive regulator of cardiac fibrosis [27], is upregulated in AF patients with mitral valve disease submitted to valve replacement, and [28] postoperative AF in patients submitted to myocardial revascularization [29]. Nevertheless, studies on aortic stenosis are scarce. MMP2, MMP9, and MMP16 showed no statistically significant differences between AF and SR patient subgroups. However, TIMP1 and TIMP2 were decreased in AF (*p* = 0.052 and *p* = 0.026, respectively), with no differences in gene expression for TIMP4. Regarding MMP/TIMP ratios, MMP2/TIMP1, MMP2/TIMP2, and MMP9/TIMP1 were similar in AF participants when compared to their SR counterparts. Conversely, MMP2/TIMP4 demonstrated a tendency towards significance, being decreased in AF patients, while MMP16/TIMP4 was significantly lower in this patient subgroup. Extracellular matrix remodeling is induced by collagen digestion through MMPs with tightly opposing inhibitors (TIMPs) [30]. Findings vary according to experimental conditions, target extracellular matrix proteins, type of cardiovascular disease, and AF progression [1]. Polyakova et al. found increased atrial expression of MMP2, MMP9, and TIMP1, although no differences in TIMP4, in AF patients submitted to a mini-Maze procedure [31]. Conversely, there were no differences in MMP9 atrial expression in AF patients with heart failure with reduced ejection fraction [32]. Despite a similar expression of TIMP4 between groups, MMP2/TIMP4 and MMP16/TIMP4 ratios demonstrated a decrease in AF patients. MMP2/TIMP4 ratio has been reported to be increased in heart failure induced by occlusion of the left coronary artery in spontaneously hypertensive rats [33]. Additionally, Wetzl et al. found a correlation between MMP2/TIMP4 ratio and mean pulmonary arterial pressure, pulmonary vascular resistance, estimated glomerular filtration rate, and tricuspid annular plane systolic excursion. Remarkably, MMP16/TIMP4 was overwhelmingly decreased in AF patients. This is the first study that suggests MMP16/TIMP4 as a marker of disease, specifically, a marker of chronic AF in aortic stenosis patients. Furthermore, although a significantly decreased atrial gene expression (TIMP1 and TIMP2) was observed, AF patients’ serum TIMP1 and TIMP2 proteins were significantly increased. This might represent a downregulation of expression when considering increased protein levels. The increase in matrix inhibitors when comparing with proteolytic enzymes (decreased MMP/TIMP ratios) may explain the collagen accumulation seen by histology, in addition to the increased mRNA collagen type III expression.

Concerning aortic stenosis severity, Aortic Valve Maximum and Mean Gradients were negatively correlated with collagen type I gene expression, collagen type I/III ratio, and serum TIMP1 protein levels. Additionally, MMP2/TIMP1 ratio was inversely correlated with aortic valve mean gradient. Aortic valve area was positively correlated with collagen type I gene expression and collagen type I/III ratio. Moreover, aortic valve area was negatively associated with cardiomyocyte cell area. Although the classical aortic valve stenosis severity definition remains dependent on transthoracic echocardiography and its parameters, such as maximal aortic velocity (severe, > 4 m/s), mean pressure gradient (severe, > 40 mmHg) and aortic valve area (severe, > 1 cm^2^), the interplay between comorbidities and aortic stenosis risk of progression remains incompletely understood [34, 35]. AF in aortic stenosis is associated with lower maximum and mean pressure gradients, which could lead to a misclassification of disease severity, since AF represents an independent risk factor for adverse outcomes, including all-cause mortality [36]. The obstruction caused by aortic stenosis leads to pressure overload and, ultimately, to ventricular hypertrophy and cardiac fibrosis [37]. Azevedo et al. found the amount of myocardial fibrosis is associated with all-cause mortality after aortic valve replacement in patients with severe aortic stenosis, indicating tissue fibrosis could be a marker of disease severity [38]. Overall, our results indicate an adverse remodelling in patients with lower maximum and mean pressure gradients, which can be explained by the presence of AF in this cohort. AF patients have worse remodelling and lower gradients, thus patients with more collagen deposition will be those with, apparently, less severe stenosis. However, a higher degree of fibrosis is associated with worse outcomes. Our results suggest several matrix proteins which could be implicated in disease severity besides histological fibrosis. Serum TIMP1 could be a marker of adverse outcomes in aortic stenosis, in addition to conventional echocardiography.

This study indicates an atrial matrix remodeling in aortic stenosis patients with chronic AF submitted to valve replacement while suggesting TIMP1 and TIMP2 as biomarkers of disease, readily available with peripheral blood sampling. MMP16/TIMP4 ratio is increased in AF and potentially a novel marker of this arrhythmia. Although the number of patients analysed is relatively low and thus further studies on chronic AF are needed, this is the first study on atrial matrix remodelling in aortic stenosis patients with atrial fibrillation.

## Conclusions

Atrial fibrillation patients with severe aortic stenosis present increased atrial fibrosis and collagen type III synthesis, with extracellular matrix remodelling demonstrated by a decrease in the MMP16/TIMP4 ratio, along with an increased serum TIMP1 and TIMP2 proteins. TIMP1 could be a marker of adverse outcomes in aortic stenosis, in addition to conventional echocardiography.

## Data Availability

The datasets used and/or analysed during the current study are available from the corresponding author on reasonable request.

## Abbreviations

AF: Atrial fibrillation
AVR: Aortic valve replacement
CAD: Coronary artery disease
CICP: Collagen type 1 carboxy-terminal peptide
DAD: Delayed afterdepolarizations
EKG: Electrocardiogram
ELISA: Enzyme-linked immunosorbent assay
MMP: Matrix metalloproteinase
NYHA: New York Heart Association
qPCR: Quantitative polymerase chain reaction
RT-PCR: Reverse transcription polymerase chain reaction
SR: Sinus rhythm
TGF-β1: Transforming growth factor β1
TIMP: Tissue inhibitor of metalloproteinase
